# Association of intraoperative transfusion of blood products with mortality in lung transplant recipients

**DOI:** 10.1186/2047-0525-2-20

**Published:** 2013-09-27

**Authors:** Denise Weber, Silvia R Cottini, Pascal Locher, Urs Wenger, Paul A Stehberger, Mario Fasshauer, Reto A Schuepbach, Markus Béchir

**Affiliations:** 1 Surgical Intensive Care Medicine, University Hospital of Zurich, Raemistrasse 100, Zurich, CH-8091, Switzerland

**Keywords:** Lung transplantation, Mortality, Transfusion, ICU complications

## Abstract

**Background:**

The impact of intraoperative transfusion on postoperative mortality in lung transplant recipients is still elusive.

**Methods:**

Univariate and multivariate analysis were performed to investigate the influence of red blood cells (RBCs) and fresh frozen plasma (FFP) on mortality in 134 consecutive lung transplants recipients from September 2003 until December 2008.

**Results:**

Intraoperative transfusion of RBCs and FFP was associated with a significant increase in mortality with odds ratios (ORs) of 1.10 (1.03 to 1.16, *P* = 0.02) and 1.09 (1.02 to 1.15, *P* = 0.03), respectively. For more than four intraoperatively transfused RBCs multivariate analysis showed a hazard ratio for mortality of 3.8 (1.40 to 10.31, *P* = 0.003). Furthermore, non-survivors showed a significant increase in renal replacement therapy (RRT) (36.6% versus 6.9%, *P* <0.0001), primary graft dysfunction (PGD) (39.3% versus 5.9%, *P* <0.0001), postoperative need of extracorporeal membrane oxygenation (ECMO) (26.9% versus 3.1%, *P* = 0.0019), sepsis (24.2% versus 4.0%, *P* = 0.0004), multiple organ dysfunction syndrome (MODS) (26.9% versus 3.1%, *P* <0.0001), infections (18.1% versus 0.9%, *P* = 0.0004), retransplantation (12.1% versus 6.9%, *P* = 0.039) and readmission to the ICU (33.3% versus 12.8%, *P* = 0.024).

**Conclusions:**

Intraoperative transfusion is associated with a strong negative influence on outcome in lung transplant recipients.

## Background

Liberal transfusion practice has been shown to exert negative influences on morbidity and mortality in different groups of patients due to increased incidence in viral and bacterial infections, activation of inflammatory and coagulation pathways, and immunologic reactions related to transfusion of red blood cells (RBCs) [1]. After lung transplantation the grafts are vulnerable for dysfunction because of denervation, postischemic condition, absent perfusion of bronchial arteries and lacking of lymphatic drainage system. In this context, transfusion-related acute lung injury (TRALI) and transfusion-associated circulatory overload (TACO) are more common than infectious complications, and increase morbidity and mortality [2]. Transfusion of fresh frozen plasma (FFP) may also be associated with adverse effects as transfusion of FFP has been independently associated with a higher risk of developing multiple organ dysfunction syndrome (MODS) and acute respiratory distress syndrome (ARDS) in trauma [3] and critically ill patients [4]. Recently, we demonstrated that intraoperative transfusion of more than 7 units of RBCs in liver transplant recipients was associated with increased mortality [5]. In lung transplant recipients, however, we still lack clear data on the influence of transfusion practice on outcome. To date, there is only one report demonstrating reduced survival after platelet transfusion in lung transplant recipients [6].

The present retrospective study was designed to investigate the influence of intraoperative transfusion of RBCs and FFPs on mortality in lung transplant recipients.

## Methods

Following approval by the local Ethics Committee of the Canton of Zurich, Switzerland, (KEK 4), which waived the need for written informed consent for this retrospective analysis, we included a total of 134 consecutive lung transplant recipients who were treated at the ICU, University Hospital of Zurich, Zurich, Switzerland. The study period was 75 months, from September 2003 until December 2008.

### Pretransplant recipient data

For baseline characteristics, we collected age, gender, height and weight. The underlying disease and the presence or absence of pulmonary hypertension (PH) was documented. Furthermore, we assessed whether the patients were admitted to transplantation from home, normal ward or an ICU.

### Intraoperative data

The number of transfused units of RBCs, FFP, platelet concentrates, fibrinogen and the use of extracorporeal circulation (ECC) during the operation were recorded. The management of coagulation and transfusions practice was done according to internal guidelines. In fact a hemoglobin threshold of 7 g/dL and a coagulation management aiming at functional fibrinogen levels as assessed by thromboelastometry were used [7].

### ICU data

The following data were collected: length of stay (LOS) in the ICU, ventilation days, occurrence of renal replacement therapy (RRT) and of primary graft dysfunction (PGD), postoperative use of extracorporeal membrane oxygenation (ECMO), occurrence of sepsis and MODS, frequency of readmission to the ICU, and need of retransplantation. Indications for ECMO were exactly as described previously [8] and for RRT the occurrence of risk, injury, failure, loss, end-stage renal disease (RIFLE) class 2 (injury), as we use routinely in our transplant programme [5,9].

### Analyzing protocol

All of the data were extracted from the patients’ charts. Endpoint of the observational period and for analysis was 31 December 2008. The patients were divided into survivors (S) and non-survivors (NS).

First, baseline and clinical data were analyzed and compared. Then, intraoperative and ICU data were analyzed and compared between the two groups.

Survival was analyzed in terms of ICU mortality and cumulative survival over the entire observation period. In addition, we performed subgroup analyses assessing cumulative survival comparing the patients that received more than four RBCs to the ones with less RBCs transfused. Finally, a multivariate analysis was done to examine whether transfusing RBCs or FFPs are independent risk factors for mortality.

### Statistical analysis

Univariate analysis was done with chi-square test for nominal data; unpaired data analysis for continuous variables was performed by means of Mann–Whitney test or logistic regression. For multivariate analysis we used a Cox proportional hazards model to identify independent risk factors for mortality. Calculation of cumulative survival overall and for the subgroups was done by Kaplan–Meier analysis with log rank testing. Data are expressed as mean ± SD; different data expression is stated in the text. All calculations were done with StatView 4.5 (Abacus Concepts, Berkeley, CA, USA). Statistical significance was accepted with *P* <0.05 (two-sided tests).

## Results

### Univariate analysis

#### Baseline characteristics in survivors and non- survivors

Baseline characteristics of the recipients are shown in Table 1. There was no difference in the incidence of PH between survivors and non-survivors (*P* = 0.16). Concomitant lung disease was similar in all patients, without any statistically significant difference between the two groups (*P* = 0.65) (Table 2). While 104 patients were admitted from home (82 survivors, 22 non-survivors), 22 were admitted from the normal wards (14 survivors, 8 non-survivors) and 8 patients from the ICU (6 survivors, 2 non-survivors), there was no difference between the two groups (*P* = 0.41). Mean donor age was 42.5 ± 14.2 years (42.1 ± 14.1 years in the survivors versus 43.8 ± 15.1 years in the non-survivors group, *P* = 0.57). Taken together, there were no statistically significant differences between survivors and non-survivors.

**Table 1:** Baseline characteristics

	**All (n = 134)**	**S (n = 102)**	**NS (n = 32)**	***P***
Men	79 (78.9%)	58 (56.9%)	21 (65.6%)	0.56
Women	55 (41.9%)	44 (43.1%)	11 (34.4%)	0.56
Age (years)	44.8 ± 16.4 (13 to 68)	44.6 ± 16.7 (13 to 68)	45.8 ± 15.8 (18 to 66)	0.71
Weight (kg)	60.6 ± 16.6 (32 to 122)	59.9 ± 15.2 (32 to 100)	62.8 ± 20.2 (37 to 122)	0.38
Height (m)	1.67 ± 0.90 (1.42 to 1.90)	1.66 ± 0.90 (1.42 to 1.90)	1.69 ± 0.90 (1.55 to 1.88)	0.14
BMI (kg/m^2^)	21.6 ± 5.0 (14.0 to 39.4)	21.6 ± 5.0 (14.0 to 36.4)	21.8 ± 6.0 (14.5 to 39.4)	0.75
Creatinine (μmol/l)	74 ± 20 (29 to 147)	74 ± 21 (29 to 147)	75 ± 18 (44 to 110)	0.86
PH (pts)	61 (45.5%)	43 (42.1%)	18 (56.2%)	0.16

Data expressed as mean ± SD (range). *BMI* body mass index, *PH* pulmonary hypertension, *pts* patients.

**Table 2:** Underlying lung diseases

	**All (n = 134)**	**S (n = 102)**	**NS (n = 32)**	***P***
CF	47 (35.0%)	38 (37.2%)	9 (28.1%)	
COPD	25 (18.6%)	19 (18.6%)	6 (18.8%)	
IPF	27 (20.1%)	19 (18.6%)	8 (25.0%)	
Alpha-1 deficiency	13 (9.8%)	12 (11.8%)	1 (3.1%)	Chi-squared test for all
Re-TPL	7 (5.3%)	5 (4.9%)	2 (6.3%)	0.65
PPH	6 (4.5%)	3 (2.9%)	3 (9.3%)	
Sarcoidosis	3 (2.2%)	2 (2.0%)	1 (3.1%)	
Others	6 (4.5%)	4 (4.0%)	2 (6.3%)	

Data expressed as absolute numbers (percentage). *CF* cystic fibrosis, *COPD* chronic obstructive pulmonary disease, *IPF* interstitial pulmonary fibrosis, *re-TPL* retransplantation, *PPH* primary pulmonary hypertension.

#### Intraoperative transfusion and use of intraoperative extracorporeal circuit

Intraoperative transfusion of RBCs and FFP was associated with a significant increase in mortality with odds ratios (ORs) of 1.10 (1.03 to 1.16, *P* = 0.02) and 1.09 (1.02 to 1.15, *P* = 0.03). There were no statistically significant differences regarding transfusion of platelets (*P* = 0.48) and application of fibrinogen (*P* = 0.90). Overall, there was no difference in the use of extracorporeal circuit between the groups (*P* = 0.31). Details are given in the Tables 3 and 4.

**Table 3:** Transfusion requirements

	**All (n = 134)**	**S (n = 102)**	**NS (n = 32)**	***P***
RBC (U)	5.8 ± 6.2 (0 to 32)	4.8 ± 5.0 (0 to 32)	8.8 ± 8.4 (0 to 29)	0.02
FFP (U)	4.1 ± 6.6 (0 to 30)	3.1 ± 5.2 (0 to 30)	7.2 ± 9.0 (0 to 29)	0.03
Platelets (10^3^/μl)	0.6 ± 1.9 (0 to 16)	0.5 ± 2.1 (0 to 16)	0.8 ± 1.5 (0 to 6)	0.48
Fibrinogen (g)	1.3 ± 2.4 (0 to 12)	1.3 ± 2.4 (0 to 12)	1.4 ± 2.3 (0 to 6)	0.90

Data expressed as mean ± SD (range). *RBC* red blood cell concentrate, *FFP* fresh frozen plasma.

**Table 4:** Intraoperative extracorporeal circuit

	**All**	**S**	**NS**	***P***
ECC (pts)	65 (48.5%)	47 (46.0%)	18 (56.2%)	0.31

Data expressed as absolute numbers (percentage). *ECC* extracorporeal circulation, *pts* patients.

#### ICU journey

In non-survivors, LOS in the ICU (*P* = 0.0007) and ventilation days (*P* = 0.011) were significantly prolonged compared to survivors. Furthermore, non-survivors had significantly higher incidence of RRT (*P* <0.0001), PGD (*P* <0.0001), postoperative ECMO (*P* = 0.002), sepsis (*P* <0.0001), MODS (*P* <0.0001), retransplantation (*P* = 0.040) and readmission to the ICU (*P* = 0.024). Details are shown in Table 5.

**Table 5:** ICU journey

	**All (n = 134)**	**S (n = 102)**	**NS (n = 32)**	***P***
LOS ICU (d)	12.9 ± 24.8 (2 to 164)	8.3 ± 10.9 (2 to 62)	27.0 ± 43.8 (2 to 164)	0.0007
LOS hospital (d)	49.2 ± 40.3 (3 to 270)	45.5 ± 29.0 (24 to 238)	60.7 ± 63.1 (3 to 270)	0.075
Ventilation (d)	9.6 ± 24.3 (1 to 162)	5.0 ± 9.1 (1 to 51)	23.9 ± 43.7 (1 to 162)	0.011
CRRT (pts)	14.2% (19)	6.8% (7)	37.5% (12)	<0.0001
PGD (pts)	14.2% (19)	5.9% (6)	40.6% (13)	<0.0001
ECMO (pts)	7.4% (10)	2.9% (3)	21.9% (7)	0.002
Sepsis (pts)	9.0% (12)	4.0% (4)	25.0% (8)	<0.0001
MODS (pts)	11.2% (15)	2.9% (3)	37.5% (12)	<0.0001
Re-TPL (pts)	8.3% (11)	6.8% (7)	12.6% (4)	0.040
Readmission ICU (pts)	17.2% (23)	11.8% (12)	24.4% (11)	0.024

Data expressed as mean ± SD (range) or percentage of incidence (number of patients). *CRRT* continuous renal replacement therapy, *d* days, *ECMO* extracorporeal membrane oxygenation, *LOS ICU* length of stay in ICU, *LOS hospital* length of stay in hospital, *MODS* multiple organ dysfunction syndrome, *PGD* primary graft dysfunction, *pts* patients, *re-TPL* retransplantation.

#### Mortality rate

**Figure 1:**
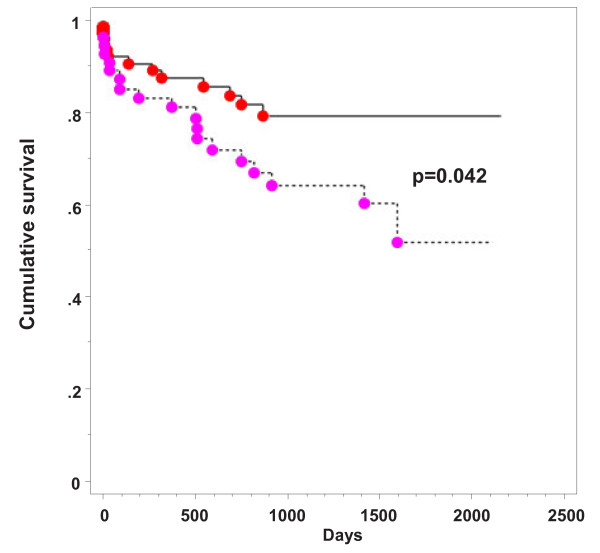
**Kaplan–Meier analysis with log rank test.** There was a significantly lower cumulative 1-year survival in the group with transfusion of more than four RBCs (dashed line). RBC, red blood cell.

Overall ICU mortality was 8.9% (12 out of 134 patients). Overall cumulative 1-year survival was 86% and cumulative 3-year survival was 72.8%. There was a significantly decreased cumulative 1-year survival in the group with transfusion of more than four RBCs (survival 81.2% versus 87.6%, *P* = 0.042). For details see Figure 1.

### Multivariate analysis

#### Risk factors for mortality

The Cox proportional hazards model for mortality identified transfusion of more than four RBCs (*P* = 0.003), sepsis in the ICU (*P* = 0.045), PGD (*P* = 0.0004) and RRT in the ICU (*P* = 0.015) as independent risk factors for mortality. In contrast, use of cardiopulmonary bypass intraoperatively (*P* = 0.35), pre-existing PH (*P* = 0.36), transfusion of more than five FFPs (*P* = 0.78), age >60 years (*P* = 0.10), RRT 6 months after transplantation (*P* = 0.39), body mass index (BMI) (*P* = 0.09) and more than 9 days of ventilation in the ICU (*P* = 0.12) were not discriminated as risk factors for mortality. Details are given in Table 6.

**Table 6:** Cox proportional hazards model for mortality (n = 134)

**Parameter**	***P***	**Hazard ratio**	**CI**
Sepsis in ICU	0.045	3.8	1.02 to 14.8
PGD	0.0004	5.4	2.1 to 13.6
Transfusion >4 RBC	0.003	4.7	1.7 to 13.3
CRRT in ICU	0.015	4.0	1.3 to 12.5
>9 ventilation days	0.12	2.2	0.8 to 6.1
BMI >25 kg/m^2^	0.087	1.07	0.99 to 1.13
Transfusion >5 FFP	0.78	1.14	0.5 to 2.6
Age >60 years	0.10	0.5	0.2 to 1.2
PH	0.36	1.5	0.7 to 3.3
RRT 6 months after TPL	0.39	2.2	0.4 to 13.5
ECC	0.35	0.7	0.3 to 1.6

*BMI* body mass index, *CI* confidence interval, *CRRT* continuous renal replacement therapy, *ECC* extracorporeal circulation, *FFP* fresh frozen plasma, *PGD* primary graft dysfunction, *PH* pulmonary hypertension, *RBC* red blood cell, *RRT* renal replacement therapy, *TPL* transplantation.

## Discussion

The main finding of this study is that intraoperative transfusions of RBCs were associated with increased mortality in lung transplant recipients.

There is a growing body of evidence that the number of intraoperative transfusions alters outcome in different patient populations. Gajic *et al.* demonstrated that 8% of transfused critically ill patients developed acute lung injury within 6 hours after transfusion [4]. In another study, Christie *et al.* showed that increased amounts of soluble receptors for advanced glycation end products (a marker for lung epithelial injury) as a consequence of transfusion was clearly associated with PGD in lung transplant recipients [10]. Furthermore, intraoperative transfusion of platelets was associated with worse outcome after lung transplantation [6]. To date, we are still lacking literature regarding the impact of transfusions on long-term outcome in lung transplant recipients. In liver transplant recipients, however, Massicotte *et al.* prospectively demonstrated that transfusion was associated with worse outcome [11].

Interestingly, in our study population there were no baseline differences between the groups; in particular, there was no influence of underlying diagnosis, intraoperative cardiopulmonary bypass use or pre-existing PH to transfusion rates, which could explain these findings. Nevertheless, we cannot exclude such a difference because of the small sample size, in which the analysis might fail revealing relevant differences between survivors and non-survivors. But, taken together, in this context our study supports the hypothesis that intraoperative transfusion might have negative impact on outcome in lung transplantation recipients. As a consequence, avoiding intraoperative transfusion of at least RBCs as much as feasible might be a beneficial strategy to improve survival in such patients.

A limitation of that suggestion and of this study is clearly the retrospective design. Other limitations are that we did not systematically measure comorbidities in our population and therefore we cannot exclude such an influence on the transfusion requirement. Therefore, a prospective approach in an equal design as Massicotte used in liver transplantation adapted to lung transplants would be important to strengthen or rule out the conclusions drawn from this retrospective setting. Furthermore, the sample size of the study is only moderate and therefore our results must be interpreted very carefully. However, as long as we lack data of a larger sample size or of prospective trials, we recommend being careful with intraoperative transfusion in lung transplantation.

## Conclusions

Intraoperative transfusion of RBCs appears to be an independent risk factor for worsened outcome in lung transplant recipients. Therefore, transfusions should be reduced and be avoided as much as possible, at least as long as we lack results of further prospective data.

## Abbreviations

ARDS: Acute respiratory distress syndrome; BMI: Body mass index; CF: Cystic fibrosis; CI: Confidence interval; COPD: Chronic obstructive pulmonary disease; CRRT: Continuous renal replacement therapy; ECC: Extracorporeal circulation; ECMO: Extracorporeal membrane oxygenation; FFP: Fresh frozen plasma; IPF: Interstitial pulmonary fibrosis; LOS: Length of stay; MODS: Multiple organ dysfunction syndrome; OR: Odds ratio; PGD: Primary graft dysfunction; PH: Pulmonary hypertension; PPH: Primary pulmonary hypertension; RBC: Red blood cell; re-TPL: Retransplantation; RIFLE: Risk, injury, failure, loss, end-stage renal disease; RRT: Renal replacement therapy; TACO: Transfusion-associated circulatory overload; TPL: Transplantation; TRALI: Transfusion-related acute lung injury.

## Competing interests

The authors declare that they have no competing interests.

## Authors’ contributions

DW, PAS and SRC performed data analysis, interpretation and drafted the article. PL and UW undertook statistics and critical review. RAS and MF undertook data collection and critical review. MB conceived the study, and undertook interpretation and critical review. All authors read and approved the final manuscript.
